# Exoscope-Assisted Spine Surgery: A Systematic Review From Basic to Complex Pathologies

**DOI:** 10.7759/cureus.88450

**Published:** 2025-07-21

**Authors:** Vivek Sanker, Vamshi Krishna DV, Prachi Dawer, Tirth Dave, Tanvi Banjan, Ahed H Kattaa, Maria Jose Cavagnaro, David J Park, Steven D Chang, Corinna Clio Zygourakis, Atman Desai, Harminder Singh

**Affiliations:** 1 Department of Neurosurgery, Stanford University School of Medicine, Stanford, USA; 2 Department of Medicine and Surgery, Institute of Medical Sciences, Banaras Hindu University, Varanasi, IND; 3 Department of Medicine and Surgery, University College of Medical Sciences, New Delhi, IND; 4 Department of Neurosurgery, Bukovinian State Medical University, Chernivtsi, UKR; 5 Department of Neurosurgery, Grant Government Medical College, Mumbai, IND; 6 Department of Neurosurgery, Stanford University Medical Center, Stanford, USA; 7 Division of Neurosurgery, Santa Clara Valley Medical Center, San Jose, USA

**Keywords:** artificial intelligence, exoscope, neurosurgery, operating microscope, spine surgery, systematic review

## Abstract

Operative neurosurgery has greatly benefited from technological advancements over the past several decades. However, challenges such as limited visualization, intraoperative navigation difficulties, and the complexity of spinal anatomy continue to pose significant hurdles for surgeons. The utilization of advanced technologies, such as exoscopes, navigation systems, and robotics, can help overcome some of these challenges, thereby enhancing surgical precision and accuracy. Over time, spine surgery has undergone remarkable advancements. Among those, exoscope-assisted spine surgery stands out as a promising approach, providing surgeons with an unmatched visual experience and enhancing the potential for improved patient outcomes. The objective of this systematic review is to examine the current use of exoscopes in spine surgery and compare the available technologies and types. We conducted a systematic review of the literature for exoscopes in spine surgeries using the Preferred Reporting Items for Systematic Reviews and Meta-Analyses (PRISMA) methodology across four major reliable databases: PubMed, ScienceDirect, Embase, and Scopus. A total of 42 studies were included in this review. We aim to present a comprehensive overview of exoscope-assisted spine surgeries, focusing on the technology's evolution, advantages, clinical applications, and potential limitations. Adequate lighting, magnification, and precision in the identification of critical surgical tissues are essential for predicting the "maximal safe resection" in neurosurgery. Vital neurovascular structures can be recognized and dissected using the high-resolution illumination provided by the operating microscope (OM). Conversely, the OM has several disadvantages, including a limited field of view, difficulty seeing around corners, and the potential to impose uncomfortable surgical postures. Additionally, an OM is difficult to maneuver around the operating room due to its size and weight. The exoscope distinguishes itself from traditional surgery by positioning the camera externally to the surgical area, providing the surgeon with an improved ergonomic vantage point to visually oversee the operative field. With more visibility, surgeons can navigate the spine and its supporting components, potentially improving treatment precision. Exoscopes offer numerous advantages over traditional OMs, including higher magnification, enhanced 3D visualization, improved ergonomics, and greater flexibility. These benefits increase precision, reduce surgeon fatigue, and enhance surgical outcomes. The use of exoscopes in spine surgery has shown promise in reducing bleeding, improving hemostasis, and potentially shortening surgical times. Additionally, the ability to record and stream surgical procedures facilitates better communication and collaboration among the surgical team, benefiting experienced surgeons and trainees. Surgeons may face a learning curve when transitioning from traditional microscopes to exoscopes, but this hurdle can be overcome with adequate training and experience. The initial high procurement costs and limited availability of exoscopes in resource-constrained areas may also pose barriers to widespread adoption.

## Introduction and background

Exoscopes represent a new technological advancement of imaging systems that help surgeons ergonomically over the conventional OM without looking directly into the interface [1,2]. The exoscope system comprises an articulated arm for focusing on the microsurgical field, a high-resolution 4K monitor, a navigation system, a hand-motion detector with electromagnetic sensors, and additional instrumentation. The exoscope provides a high-definition 3D digital camera system with enhanced ergonomics [1], increased mobility, and improved collaboration among the surgical team. Integration with navigation systems further enhances accuracy and adaptability, leading to better patient outcomes, shorter recovery times, and an overall improved quality of life for individuals with spinal conditions and injuries [3,4].

Before the exoscope, the introduction of an operating microscope (OM) was a significant milestone in the field of neurosurgery. OM enabled surgeons to magnify and illuminate the surgical field, thereby allowing for precise and intricate procedures that were previously challenging or impossible to perform with conventional eyesight alone [5]. However, methods of visualization and optics of OM have reached a plateau. Additionally, limitations such as limited space and mobility [6], a narrow depth of field, and a limited focal length require the spinal surgeon and first assistant to directly contact the OM objective, which ultimately leads to physical strain and discomfort. This nonergonomic posture is linked to work-related musculoskeletal injuries among surgeons [7,8]. Exoscope-assisted spine surgery is a promising approach, offering surgeons an unparalleled visual experience and enhancing the potential for improved patient outcomes.

Over several years, exoscopes like ORBEYE™ (Olympus, Tokyo, Japan), BrainPath® (Nico Corporation, Indianapolis, IN, US), Modus V™ (Synaptive Medical, Toronto, ON, Canada), VITOM® 3D (Karl Storz, Tuttlingen, Germany), Aeos® (Aesculap, Tüttlingen, Germany), and KINEVO 900 S (Carl Zeiss Meditec AG, Jena, Germany) are developed. As with any evolving technology, exoscope-assisted spine surgery may present limitations, such as cost, equipment availability, the need for specialized training, and a learning curve.

While numerous review articles have explored the advantages of exoscopes in neurosurgery compared to traditional OMs [2,6,9], further research is still needed to examine their potential benefits and limitations in the context of spine surgical procedures. This systematic review article aims to present a comprehensive overview of exoscope-assisted spine surgeries, exploring the technology's evolution, advantages, clinical applications, and potential limitations. By exploring the latest advancements in exoscope technology and its clinical applications, we hope to inspire further research, foster informed decision-making, and ultimately optimize patient care in spine surgery.

## Review

Methods

**Figure 1:**
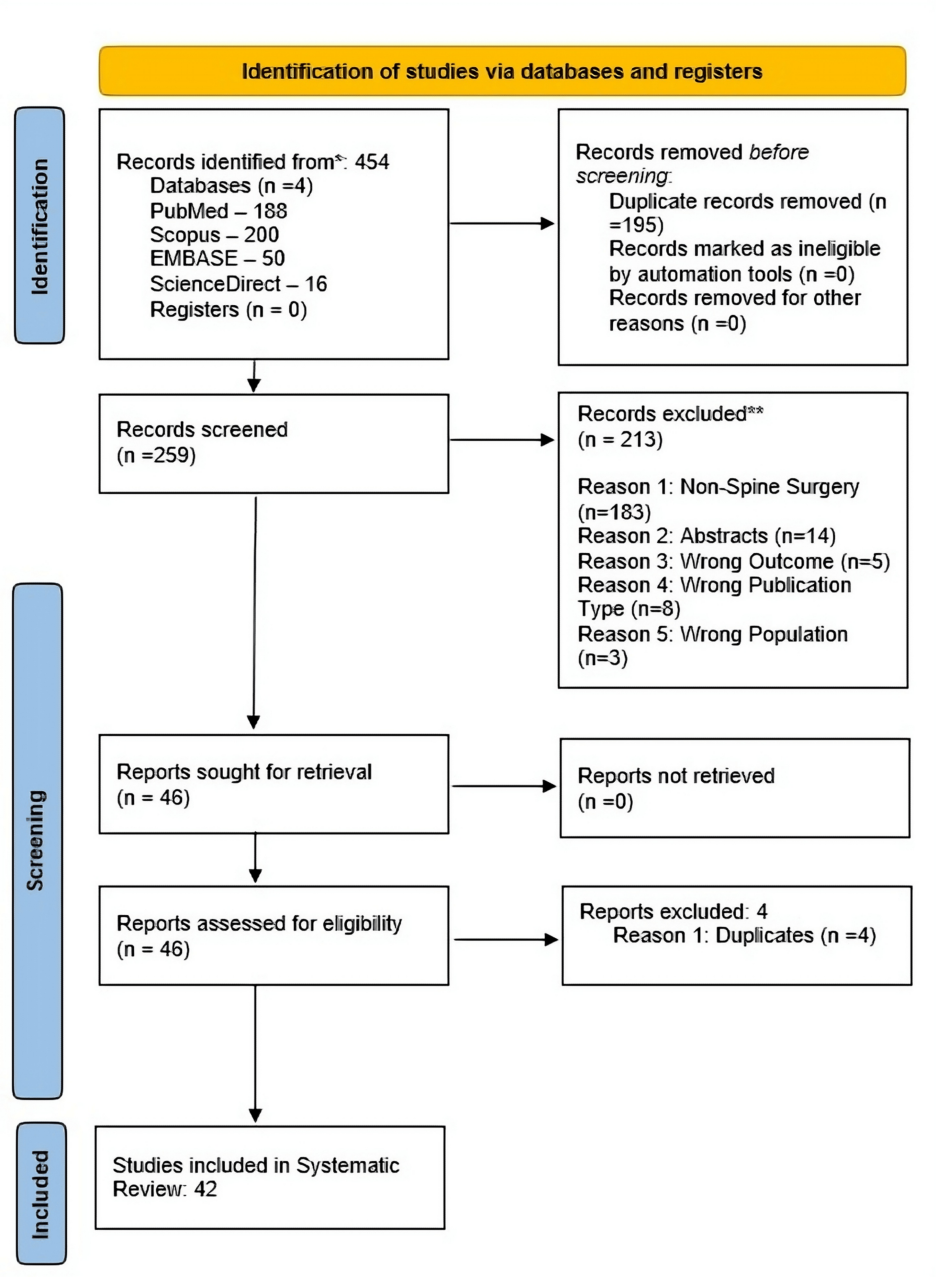
PRISMA flowchart for the literature screening and selection process PRISMA: Preferred Reporting Items for Systematic Reviews and Meta-Analyses

The present systematic review adheres to the Preferred Reporting Items for Systematic Reviews and Meta-Analyses (PRISMA) guidelines. Four major databases, Scopus, PubMed, ScienceDirect, and Embase, were utilized to gather relevant papers. A total of 42 papers were included and referenced after undergoing a rigorous screening process that excluded irrelevant studies. The detailed screening and selection process is illustrated in Figure 1.

The search strategy employed the following terms: exoscope, spine, microsurgery, spine surgery, spinal microsurgery, minimally invasive surgery, and minimally invasive surgical procedures. Table 1 presents the exact search strategies employed to gather the required literature.

**Table 1:** Search terms used to obtain required literature from selected databases

Database	Search string	Results
Scopus	(TITLE-ABS (exoscope)) AND ((spine) OR (Microsurgery) OR ("spine surgery") OR ("Spinal microsurgery") OR ("Minimally Invasive Surg*") OR (INDEX TERMS ("Minimally Invasive Surgical Procedures")))	200
PubMed	(Exoscope [Title/Abstract]) AND ((spine) OR (Microsurgery) OR (spine surgery) OR (Spinal microsurgery) OR (Minimally Invasive Surg*) OR (Minimally Invasive Surgical Procedures [MeSH]))	188
ScienceDirect	Exoscope, Spine surgery	16
Embase	(exoscope.tw.) AND ((spine) OR (Microsurgery) OR ("spine surgery") OR ("Spinal microsurgery") OR ("Minimally Invasive Surg*") OR (exp "Minimally Invasive Surgical Procedures"/))	50

This approach aimed to identify articles that discussed the use of exoscopes in spine surgeries, excluding those that focused on exoscopes in surgeries other than spine surgeries. The inclusion criteria for the selected articles were specific to their relevance to the use of exoscopes in spine surgeries. Any papers that mentioned exoscope applications in surgical procedures unrelated to the spine were excluded from consideration.

Two authors reviewed the titles and abstracts of potentially relevant articles during the screening process. Furthermore, the cited references of each publication were scrutinized to identify additional pertinent information. In cases where conflicts arose concerning whether a manuscript should be included, a third author was consulted to reach a consensus. Data from each eligible study were extracted and compiled in an Excel spreadsheet, facilitating the synthesis of a comprehensive review.

Results

Our systematic review included 42 articles after a thorough literature search from 4 databases (PubMed, Embase, Scopus, and ScienceDirect). The list of articles, along with key findings, is summarized below (Table 2).

**Table 2:** Characteristics and key findings of studies included in the systematic review on exoscope-assisted spine surgery ACDF: anterior cervical discectomy and fusion, ACF: anterior cervical foraminotomy, ALT: anterolateral thigh, COVID-19: coronavirus disease 2019, CSM: cervical spondylotic myelopathy, CVST: cerebral venous sinus thrombosis, EMIS-TLIF: exoscope-assisted minimally invasive transforaminal lumbar interbody fusion, ENT: ear, nose, and throat, FCP: foot control pedal, HD: high definition, HMD: head-mounted display, JOA: Japanese Orthopedic Association, KPS: Karnofsky performance status, LDD: lumbar degenerative disease, LPD: lumbar posterior decompression, MIS: minimally invasive surgery, MIS-TLIF: minimally invasive transforaminal lumbar interbody fusion, N/A: not applicable, NASA-TLX: National Aeronautics and Space Administration task load index, NDI: neck disability index, OM: operating microscope, OMIS-TLIF: operating microscope-assisted minimally invasive transforaminal lumbar interbody fusion, OR: operating room, PPE: personal protective equipment, PGY: postgraduate year, RDM: robotic digital microscope, REBA: rapid entire body assessment, TLIF: transforaminal lumbar interbody fusion, VAS: visual analog scale, VTE: venous thromboembolism, 2D: two-dimensional, 3D: three-dimensional

Author, year	Study design	Sample size	Specific procedures	Key findings	Outcomes	Unfavorable occurrences	Conclusions
Lin, 2023 [10]	Prospective comparative cohort	90 patients (47 exoscope, 43 microscope)	MIS-TLIF for lumbar disc herniation	Exoscope improved ergonomics (REBA score: 5.0 vs. 6.05, p=0.017) and reduced musculoskeletal strain. Image quality and depth perception were inferior to those of the microscope in deep approaches	Comparable operative time, blood loss, and clinical outcomes (VAS, ODI) between groups	Lack of stereopsis; occasional glare; 3D glasses caused discomfort (headache/eye fatigue)	The exoscope was a safe and effective alternative to OM for assisting the MIS-TLIF procedure, with the unique advantage of excellent ergonomics to reduce musculoskeletal injuries
Ramirez et al., 2023 [11]	Prospective comparative trial	26 patients (13 exoscope, 13 microscope)	ACDF for cervical myelopathy	A low-cost exoscope ($150) was non-inferior to the microscope in terms of safety/usability. Superior for teaching (80% agreement). Inferior image quality/illumination	No complications; similar operative time/blood loss	No stereoscopic vision; cumbersome camera adjustment; limited zoom functionality	The combined use of a dedicated microsurgical robot and exoscope imaging results in enhanced visualization, precision, and ergonomics for (micro)surgeons. These benefits will result in improved patient outcomes and also decrease the number of musculoskeletal disorders for medical staff
Ramirez et al., 2023 [12]	Feasibility study	16 patients (8 exoscopes, 8 microscopes)	Open/MIS-TLIF for lumbar degenerative disease	Exoscope is a feasible option for TLIF, yielding comparable outcomes. Rated superior for teaching (60% agreement)	No complications; equivalent blood loss/operative time	Inferior brightness (70% of users); shallow learning curve due to lack of 3D	A low-budget exoscope is safe and feasible for use in TLIF. It is of significant benefit in surgical teaching. Yet, it is purchasable at a significantly lower price than conventional microscopes
Van Mulken et al., 2023 [13]	Case report	1 patient	Free ALT flap for tibial nonunion (microsurgical anastomosis)	First integrated use of robotic exoscope (MUSA + ORBEYE) enabled precise anastomoses with enhanced ergonomics	Successful flap survival; uneventful recovery	High cost, time-consuming setup, limited to high-resource settings	The low-cost exoscope appears to be a safe and effective alternative for OM-assisted ACDF with great comfort and ergonomics, and serves as an essential tool for education and training purposes
Peron et al., 2023 [14]	Case report	1 patient	C1-C2 meningioma resection	4K-3D exoscope provided superior ergonomics and visualization for intradural tumor dissection	Complete tumor resection; resolved hemiparesis	Frequent camera adjustments needed; steep learning curve for high-magnification work	The 4K-3D exoscope offers many advantages in spinal tumor surgery, such as excellent image quality with HD of anatomical details and the possibility for surgeons to operate in a comfortable position
Motov et al., 2022 [15]	Prospective observational	17 patients	Spinal surgeries (degenerative, tumor, infectious cases)	High satisfaction with image resolution, 3D depth perception, and ergonomics. Improved surgical corridor and reduced fatigue. Setup conflicts with additional equipment (e.g., fluoroscopy)	88% of surgeons rated their satisfaction as high/very high. No system-specific complications	Hand-eye coordination is affected in 11% of cases. The setup conflicts with the monitor positioning	N/A
Chebib et al., 2023 [16]	Prospective cohort	151 surgeries	Pediatric ENT surgeries (otologic, transoral, head/neck)	Superior ergonomics and educational benefits. High effectiveness in transoral and head/neck surgeries. Pixelization at high magnification in otologic surgeries	Mean scores: 92.9/100 (transoral), 89.5/100 (head/neck). No intraoperative complications	One case of eye strain led to switching to a microscope. Challenges in bilateral simultaneous surgeries (e.g., cochlear implants)	3D-exoscope appears to be a relevant tool for pediatric head and neck surgery, applicable in otologic, transoral, and cervical fields. It presents educational and ergonomic advantages and improves surgical team communication
Yao et al., 2022 [17]	Retrospective Cohort	47 patients	EMIS-TLIF (exoscope-assisted) vs. OMIS-TLIF (Microscope-assisted) for LDD	EMIS-TLIF had a shorter operation time (111.00 ± 19.87 min) vs. OMIS-TLIF (121.92 ± 16.92 min). Lower VAS back pain and ODI scores at 1-week post-op in EMIS-TLIF group. Comparable image quality	Good-to-excellent outcomes: 90.91% (EMIS-TLIF) vs. 88.00% (OMIS-TLIF). Significant improvement in VAS and ODI scores post-op in both groups. Lower complication rates in EMIS-TLIF (9.09% vs. 12.00%)	Discomfort from 3D glasses (rated 2.80/5). Small sample size. Short-term follow-up. Subjective ergonomic assessment	EMIS-TLIF is a safe, effective alternative to OMIS-TLIF, offering shorter operation time and better ergonomics, but 3D glasses discomfort and technical limitations need addressing
Rechav Ben-Natan et al., 2022 [18]	Technical case report	1 patient	Schwab grade 5 osteotomy for thoracolumbar kyphosis	Enhanced visualization of the ventrolateral dura. Improved team coordination via shared 3D view	Successful deformity correction. No complications	Exoscope positioning required careful planning to avoid instrument obstruction	The exoscope's ergonomic design and shared viewing capability represent a valuable advancement in spine surgery, offering improved outcomes for complex deformities while facilitating education and real-time coordination among the surgical team
Abramovic et al., 2022 [19]	Cross-sectional (workshop)	34 neurosurgeons	Microsurgical training exercise (simulated)	Improved ergonomics (neutral posture). High satisfaction (80%) and image quality (82%). Steeper learning curve for younger surgeons and gamers	Median bulls-eye score: 27/30. 88% felt confident using the device	HMD weight (500 g) caused discomfort. Dizziness/nausea due to misalignment. Technical assistance is needed in 12.5% of cases	The exoscope excelled in usability, image quality, as well as in ergonomic and favorable posture, and could thus become an alternative to conventional microscopes due to the potentially elevated surgeons' comfort
Barbagallo et al., 2019 [20]	Case series	2 patients	ACDF	Comparable to microscopes. Enhanced didactic capabilities via 3D video recording. Smaller size improved maneuverability	Successful decompression and fusion. Improved OR staff involvement with 3D screens	None reported, but technical familiarity is required to avoid delays	NA
Gabrovsky et al., 2022 [21]	Pilot study (prospective)	41 patients (16 cranial, 25 spinal)	Cranial (tumors, decompressions), spinal (microdiscectomy, trauma, tumors)	Transition from microscope to RDM is feasible; NASA-TLX scores improved after 20 cases. Ergonomics improved	High performance (80%+) achieved after 20 ops. Spinal cases adapted faster (9 ops)	Initial frustration and effort are higher; the learning curve is steeper for cranial cases	After approximately 20 cranial operations, a Performance level above 80% could be reached. This transition occurred faster with spinal procedures
Das et al., 2022 [22]	Comparative (prospective)	14 pediatric cases (9 cranial, 5 spinal)	Cranial (superficial tumors), spinal (myelomeningocele, tumors)	2D-VITOM is suitable for spinal/superficial cranial cases; poor depth visualization in deep cranial cases	Spinal cases completed successfully; 7/9 cranial cases switched to microscope	Poor image quality in deep cranial cavities; bleeding sources are hard to visualize	The 2D-VITOM exoscope is suitable for most spine procedures; however, it is best reserved for less complex cranial surgeries. Less space occupying and superior ergonomics were frequently stated advantages over the OM
Giorgi et al., 2023 [23]	Case series (retrospective)	10 thoracolumbar burst fractures	Minimally invasive corpectomy with exoscope-assisted decompression	Exoscope provided better ergonomics, image definition, and reduced blood loss vs. the microscope	Reduced surgical time (155 min vs. 177 min) and blood loss (403 mL vs. 421 mL)	None reported; microscopes were not needed for any case	The stereoscopic vision provided by 3D images seems to be crucial in hand-eye coordination. There are clear advantages in terms of maneuverability, a wide field of view, deep focus, and a more comfortable posture for the spinal surgeon
Encarnacion Ramirez et al., 2022 [24]	Technical report (prospective)	10 spinal cases	TLIF via Wiltse paraspinal approach	Low-cost exoscope ($350) effective for lumbar microdiscectomy; improved over prior prototype	Pain relief in all patients; 9/10 full sensorimotor recovery	Lack of 3D vision; limited storage for recordings	The presented low-cost exoscope proved effective in lumbar microdiscectomy as part of the Wiltse paraspinal approach. It is superior compared to the initial prototype concerning affordability, image quality, and adjustability of position and angle
De Jesus Encarnacion Ramirez et al., 2022 [25]	Prospective study	16 patients	13 spinal and 3 cranial surgeries (e.g., spinal arachnoid cyst, meningioma, aneurysm clipping)	Low-cost exoscopes provided similar magnification and illumination to conventional microscopes	Successful surgical outcomes in all cases; improved ergonomics and training utility	Lack of stereoscopic view, insufficient lighting in deep corridors, cumbersome zoom adjustment	The low-cost exoscope is feasible for spinal surgeries and enhances access to micro-neurosurgical care in low-resource settings
Abunime et al., 2022 [26]	Retrospective cohort	41 patients (29 cranial, 12 spinal)	Cranial 70.7% (tumors, vascular), spinal 29.3% (decompression/fusion)	HD-2D exoscope feasible for most cases; microscope needed in 4 cranial cases for deep lesions	Gross total resection in 62.1% cranial cases; improved KPS scores	3 complications (VTE, CVST) in the cranial group; 2 mortalities	The HD-2D stereotactic exoscope offers a broader field of view, greater mean focal distance, enhanced ergonomics, and immersive stereotactic visual experience. The lack of stereopsis remains the principal limitation of its use, and further optimization of surgical outcomes might be achieved with newer 3D models
Yao et al., 2021 [17]	Retrospective cohort	48 patients	ACDF	Exoscope-assisted ACDF resulted in shorter operative time and fewer complications	Similar clinical outcomes between the exoscope and OM groups. Improved VAS and JOA scores	Dysphagia and transient hoarseness were reported in the OM group	Exoscope-assisted and OM-assisted ACDF resulted in similar clinical outcomes for CSM, while exoscope-assisted surgery may be related to a short operative time and fewer complications
Maurer et al., 2021 [27]	Prospective study	19 procedures	Cranial (12), spinal (6), and peripheral nerve (1) surgeries	Aeos exoscope provided high surgical satisfaction and improved ergonomics	No intraoperative complications. High usability and comfort reported	Suboptimal image quality and depth of field noted. Headaches and eye strain in some cases	The Aeos 3D robotic digital microscope appears feasible for safe use in a wide range of microsurgical procedures in neurosurgery. Surgical satisfaction was ranked high among the majority of neurosurgeons in our study
Lin et al., 2022 [28]	Retrospective cohort	50 patients	Single-level ACDF	Exoscope offered excellent comfort and ergonomics, comparable to OM	Similar operative times and blood loss between groups. Improved pain scores	Inferior visualization in deep areas; discomfort from 3D glasses	Exoscope appears to be a safe alternative for common ACDF, with the unique advantage of excellent comfort, and also serves as a valuable educational tool for the surgical team
D'Ercole et al., 2020 [29]	Retrospective cohort	9 patients	ALIF	Exoscope provided unobstructed access and good ergonomics in deep surgical fields	Satisfactory pain relief and radiological outcomes. Short hospitalization	Cumbersomeness in repositioning and refocusing	The instrument had dimensions and a long working distance, superior to those of an endoscope and comparable to those of an OM, which showed clear advantages in maneuverability. Moreover, the stereoscopic vision provided by 3D images proved to be crucial in hand-eye coordination
Ariffin et al., 2020 [30]	Prospective observational	69 patients	Tubular microdiscectomy, decompression, MIS TLIF, OLIF	Short learning curve (6-9 cases), improved ergonomics, reduced operating time	Symptomatic improvement, no neurological deficits	Dural tears (4 cases), OR setup rearrangement required	Exoscope is effective with a short learning curve and comparable complications to the microscope
Muhammad et al., 2019 [31]	Clinical trial	8 procedures	Spinal (cervical discectomy, laminectomy) and cranial (meningioma, schwannoma)	Better visual quality, improved surgeon comfort	Comparable to a microscope, no significant complications	Lack of depth perception, learning curve	Safe for spinal and cranial surgery, but requires 3D improvement
Kwan et al., 2019 [32]	Retrospective analysis	10 patients	ACDF, cervical corpectomy, lumbar laminectomy	Excellent visualization, ergonomic benefits	No complications, immersive surgical experience	Increased scope adjustments, longer operative time	Feasible for spinal surgery with potential for improved ergonomics
Mamelak et al., 2010 [33]	Clinical trial	16 patients	Craniotomies, spinal procedures, and neurostimulator placement	HD image quality, ease of manipulation	Comparable to a microscope for many procedures	Lack of stereopsis, cumbersome scope holder	Suitable for spinal surgery, but needs refinement in the scope holder and 3D
Peng et al., 2022 [34]	Retrospective case-control	74 patients	One- and two-level TLIF	Reduced perioperative bleeding in two-level TLIF	Improved hematological parameters, no difference in clinical outcomes	None reported	3D exoscope is a suitable alternative to a microscope
Montemurro et al., 2022 [9]	Systematic review	1711 cases	Brain tumor, skull base surgery, aneurysm clipping, cervical/lumbar spine surgery	Exoscope is safe and effective, with advantages in ergonomics and visualization	Complication rate: 2.6%; switch to OM rate: 5.8%	Limited depth perception, high cost, lack of 5-ALA use	Exoscope is a viable alternative to OM, with the potential to revolutionize neurosurgery
Bai et al., 2021 [35]	Retrospective study	19 patients	ACDF combined with ACF for cervical spondylotic radiculopathy	HD 3D exoscope improved surgical precision and outcomes	Significant improvements in JOA, NDI, VAS scores; no complications	None reported	ACDF + ACF with 3D exoscope is effective and safe for bony foraminal stenosis
Schupper et al., 2023 [36]	Multicenter survey study	155 cases	Cranial (72%) and spinal (28%) procedures, including tumor resections and laminectomies	Exoscope reduced surgeon neck and back pain significantly	Less pain reported; no conversions to microscope	Rare headaches/nausea from 3D glasses	Exoscope improves ergonomics and reduces surgeon discomfort, enhancing career longevity
Keric et al., 2022 [37]	Prospective cohort study	16 patients	Cranial and spinal micro-neurosurgery	Exoscope superior in magnification and ergonomics; OM better in image contrast/quality	No difference in NASA-TLX workload; beginners had higher task burden	Switching to OM in 3 cases due to image contrast	Exoscope is a viable alternative to OM with ergonomic benefits
De Divitiis et al., 2020 [38]	Review and illustrative case	N/A	Spinal meningioma surgery	Exoscope feasible for spinal procedures; ergonomic benefits	Safe and efficient for spinal meningioma removal	Limited by tumor size and surgeon experience	Exoscope is suitable for spinal surgery, but may require OM in complex cases
Siller et al., 2020 [6]	Prospective cohort study	60 patients	ACDF (20), LPD (40)	Exoscope safe; comparable operative times and outcomes to OM	Improved ergonomics; similar clinical outcomes	Inferior visualization in long approaches	Exoscope is a safe alternative with ergonomic advantages
Shirzadi et al., 2012 [39]	Prospective cohort study	48 patients	Lumbar decompression, TLIF	Exoscope provided outstanding image quality, comparable to OM	No difference in operative time or complications	Lack of stereopsis noted	Exoscope is a practical and economical alternative to OM
Murai et al., 2019 [40]	Observational study	22 patients	Tumor resection, aneurysm clipping, vascular anastomosis, laminectomy	Exoscope ergonomic, 4K imaging, precise for fluorescence	No complications; comfortable posture for surgeons	Eyestrain for assistants; limited range of motion	Exoscope is not suitable for all surgeries, but it offers ergonomic advantages
Oertel et al., 2017 [41]	Case series	16 patients	5 cranial, 11 spinal (e.g., microvascular decompression, tumor resections, ACDF)	Excellent instrument handling, comfort, and image quality comparable to OM	Safe and effective for spinal and less demanding cranial procedures	None related to exoscope use	Exoscope is a viable alternative to OM with superior comfort and teaching potential
Krishnan et al., 2017 [42]	Technical note	18 patients	Lumbar/cervical decompressions, discectomies, tumor resections, microsutures	HD exoscope provided excellent illumination and magnification	All procedures were completed	Cumbersome repositioning; longer operative times initially	Exoscope is effective, but requires technical improvements for wider adoption
Moisi et al., 2017 [43]	Cadaveric study	11 residents	Unilateral laminotomies	No difference in operative time or decompression quality between OM and exoscope	Exoscope is rated as more comfortable and better for teaching	Variability in evaluator grading; one outlier in the exoscope group	Exoscope is a valid alternative to OM with ergonomic and teaching advantages
Barbagallo et al., 2019 [20]	Case report	2 patients	2-level ACDF	3D exoscope provided comparable visualization to OM	Successful decompression and fusion; improved teaching capabilities	None reported	3D exoscope is a safe and effective alternative to OM in ACDF
Teo et al., 2020 [44]	Case series	8 patients	Various spinal procedures (e.g., discectomies, decompressions, tumor resection)	Exoscope compatible with PPE; improved team visualization	Comparable outcomes to OM; no complications	Initial setup time is longer; cost-prohibitive	Exoscope is beneficial during COVID-19, offering safety and workflow advantages
Roethe et al., 2020 [45]	Two-phase prospective-randomized clinical evaluation	20 (randomized), 29 (total)	Supratentorial brain tumor resections, head and spine surgeries	Improved ergonomics in monitor mode; favorable for cranial tumor surgery; hand-eye coordination requires familiarization	No significant added surgical time; ergonomics improved in monitor mode	Image quality rated inferior to optic visualization; FCP conflicts; lateral camera inclination affected hand-eye coordination	Exoscopic interventions are feasible for cranial tumor surgery but require improvements in image quality and controls
De Divitiis et al., 2019 [46]	Case-control study	9 (exoscope), 9 (control)	Lumbar discectomies	Longer operative time with exoscope, but no significant difference in postoperative outcomes	Symptomatic improvement in both groups; no neurological deficits	Longer operative time: minor setup adjustments needed	Exoscope is a valuable tool for spinal procedures, but it does not replace the microscope
Poser et al., 2024 [47]	Prospective cohort, mixed methods	16 residents (test cohort), 6 PGY-1 residents (learning curve cohort)	Lumbar herniated disc surgery using a non-cadaveric spine simulator (real spine L4/L5)	Simulator training improved mental concept and microsurgical performance by 30%. The exoscope group showed slightly better consolidation of mental concepts. High acceptance among residents (median score: 8/10)	Completion rate of herniated disc removal increased from 50% to 100%. Significant improvement in self-assessed skills (Likert scale: 1.3 to 3.3). Reduced tutor interventions over sessions	Minor interventions (e.g., incorrect instrument handling) persisted. Mental concept scores declined after a 7-day training pause.	Non-cadaveric spine simulator training is practical for neurosurgical residency, especially in the early stages. Exoscope and 3D teaching show promise for enhanced learning

Quality Assessment

The evaluation of study quality and risk of bias was conducted using the ROBINS-I tool, which examines several domains of bias, including confounding bias, selection bias, classification of interventions, deviations from intended interventions, missing data, outcome measurement, and reporting bias, among others [48]. In addition, for case reports included in the review, the CARE guidelines for observational studies were applied to assess the completeness and transparency of reporting, covering key elements such as patient demographics, clinical history, diagnostic assessments, interventions, outcomes, adverse events, and key lessons. Each article was screened twice; no article was excluded following these assessments (see: https://www.care-statement.org/) (Table 3).

**Table 3:** ROB assessment ROB: risk of bias

Article no.	Author	D1: confounding	D2: selection bias	D3: classification of intervention	D4: deviations from intended interventions	D5: missing data	D6: outcome measurement	D7: reporting bias	Overall ROB
1	Lin et al., 2023 [10]	Serious	Moderate	Low	Low	Low	Moderate	Moderate	Serious
2	Ramirez et al., 2023 [11]	Serious	Moderate	Low	Low	Low	Moderate	Moderate	Serious
3	Ramirez et al., 2023 [12]	Serious	Moderate	Low	Low	Low	Serious	Moderate	Serious
4	Motov et al., 2022 [15]	Moderate	Serious	Low	Low	Moderate	Serious	Moderate	Moderate
5	Chebib et al., 2023 [16]	Moderate	Low	Low	Low	Low	Moderate	Moderate	Moderate
6	Yao et al., 2022 [17]	Moderate	Low	Low	Low	Low	Moderate	Moderate	Moderate
7	Abramovic et al., 2022 [19]	Serious	Moderate	Low	Moderate	Low	Moderate	Serious	Serious
8	Barbagallo et al., 2019 [20]	Serious	Moderate	Low	Low	Low	Serious	Moderate	Moderate to serious
9	Gabrovsky et al., 2022 [21]	Serious	Serious	Low	Low	Moderate	Moderate	Moderate	Moderate to serious
10	Das et al., 2022 [22]	Serious	Low	Low	Moderate	Low	Serious	Serious	Serious
11	Giorgi et al., 2023 [23]	Moderate	Low	Low	Low	Low	Moderate	Moderate	Moderate
12	Encarnacion Ramirez et al., 2022 [24]	Serious	Serious	Moderate	Low	Moderate	Moderate	Moderate	Moderate to serious
13	De Jesus Encarnacion Ramirez et al., 2022 [25]	Serious	Serious	Moderate	Low	Moderate	Moderate	Moderate	Moderate to serious
14	Abunimer et al., 2022 [26]	Serious	Moderate	Low	Low	Moderate	Moderate	Moderate	Moderate to serious
15	Maurer et al., 2021 [27]	Serious	Moderate	Low	Moderate	Moderate	Moderate	Moderate	Moderate to serious
16	Lin et al., 2022 [28]	Serious	Moderate	Moderate	Low	Moderate	Serious	Moderate	Serious
17	D’Ercole et al., 2020 [29]	Moderate	Serious	Low	Low	Moderate	Moderate	Moderate	Moderate to serious
18	Ariffin et al., 2020 [30]	Serious	Moderate	Low	Low	Moderate	Moderate	Moderate	Moderate to serious
19	Muhammad et al., 2019 [31]	Serious	Moderate	Low	Low	Low	Moderate	Moderate	Moderate to serious
20	Mamelak et al., 2010 [33]	Serious	Moderate	Low	Low	Low	Moderate	Moderate	Moderate to serious
21	Peng et al., 2022 [34]	Serious	Moderate	Low	Low	Low	Moderate	Moderate	Moderate to serious
22	Bai et al., 2021 [35]	Serious	Moderate	Low	Low	Low	Moderate	Moderate	Moderate to serious
23	Schupper et al., 2023 [36]	Serious	Serious	Low	Low	Low	Serious	Moderate	Serious
24	Keric et al., 2022 [37]	Serious	Moderate	Low	Low	Low	Moderate	Moderate	Moderate to serious
25	Siller et al., 2020 [6]	Serious	Moderate	Low	Low	Low	Moderate	Moderate	moderate to serious
26	Shirzadi et al., 2012 [39]	Moderate	Low	Low	Low	Low	Moderate	Moderate	Moderate
27	Murai et al., 2019 [40]	Serious	Moderate	Low	Low	Low	Moderate	Moderate	Serious
28	Krishnan et al., 2017 [42]	Serious	Moderate	Low	Low	Low	Moderate	Moderate	Serious
29	Moisi et al., 2017 [43]	Serious	Moderate	Low	Low	Low	Moderate	Moderate	Serious
30	Roethe et al., 2020 [45]	Moderate	Low	Low	Low	Low	Moderate	Moderate	Moderate
31	De Divitiis et al., 2019 [46]	Serious	Moderate	Low	Low	Low	Moderate	Moderate	Serious
32	Poser et al., 2024 [47]	Moderate	Low	Low	Low	Low	Moderate	Moderate	Moderate
33	Yao et al., 2021 [17]	Moderate	Low	Low	Low	Low	Moderate	Moderate	Moderate
34	Van Mulken et al., 2023 [13]	Yes	Yes	Yes	Yes	Yes	Yes	Yes	Yes
35	Peron et al., 2023 [14]	Yes	Yes	Yes	Yes	Yes	Yes	No	Yes
36	Rechav Ben-Natan et al., 2022 [18]	Yes	Yes	Yes	Yes	Yes	Yes	No	Yes
37	Barbagallo et al., 2019 [20]	No	No	No	No	Yes	No	No	Yes
38	Kwan et al., 2019 [32]	No	No	No	No	Yes	Yes	No	Yes
39	De Divitiis et al., 2020 [38]	No	No	No	No	Yes	No	No	Yes
40	Oertel et al., 2017 [41]	Yes	No	No	No	Yes	No	Yes	Yes
41	Teo et al., 2020 [44]	Yes	No	No	No	Yes	Yes	Yes	Yes
42	Montemurro et al., 2022 [9]	Yes	No	No	No	Yes	Yes	Yes	Yes

Typical Setup

Exoscopes are telescope-based visualization devices that provide high-quality video imaging with an expansive field of view and considerable focus distance. The fact that exoscopes are placed far from the surgical field, at a distance of roughly 25 to 30 cm, gives them an edge over current microscopes [49]. In contrast to the endoscopic approach, the exoscope enables tools to pass beneath the scope without the need for special apparatus. Due to the relatively uniform depth of exposure, all procedures can be conducted with little to no requirement for repositioning and focusing at higher magnifications by putting the exoscope camera at the start of the surgical approach, approximately 35-40 cm above the operational region.

**Figure 2:**
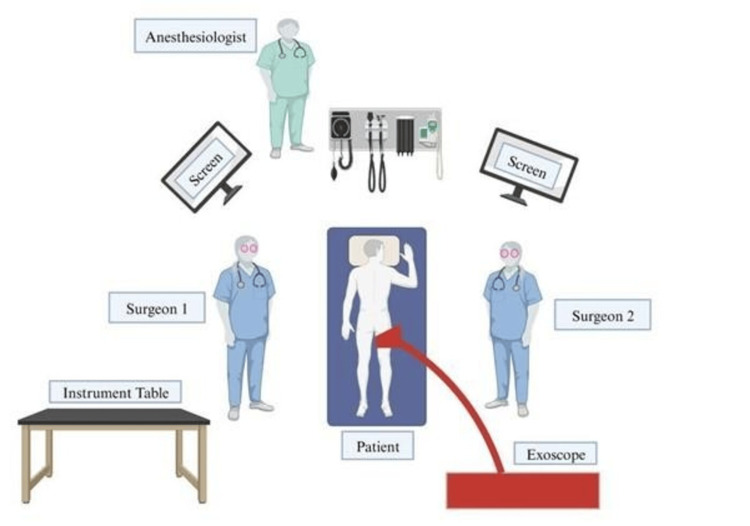
Operative setup using an exoscope for spinal procedures Image Credit: Authors. Created using BioRender.

The placements of the various surgical equipment used in each case are depicted schematically in Figure 2. To prevent visual obstructions, the chief surgeon and the assistant stood on the patient's right and left sides, respectively, and were somewhat offset. On the patient's caudal end, the exoscope is placed. The two 3D high-definition displays, positioned separately on either side of the operating table at eye level, were spaced approximately 3 feet apart and received the images produced by the camera, providing the surgical team with exceptional visualization.

Surgeons and operating room personnel utilize 3D glasses to improve depth perception during the procedure. The various exoscope systems provide the best solutions for this need (Table 4). The surgeon could quickly pull the exoscope out of the field and reposition it, allowing for frequent use of the system's high-magnification view. Similarly, leaving the exoscope in place over the operative field while placing spinal equipment was frequently possible. More practice with this approach may lead to quicker operating times and improved adaptability compared to the OM [1].

**Table 4:** Current FDA-approved systems 2D: two-dimensional, 3D: three-dimensional, 4D: four-dimensional, FDA: Food and Drug Administration, HD: high definition, ICG: indocyanine green, LED: light-emitting diode, N/A: not applicable, QEVO: quick endoscopic visualization option, WD: working distance

Platform	VITOM 3D	KINEVO 900 S	Modus V	ORBYE	Aeos
Company	Karl Storz, Tuttlingen, Germany	Carl Zeiss Meditec AG, Jena, Germany	Synaptive Medical, Toronto, ON, Canada	Olympus, Tokyo, Japan	Aesculap, Tüttlingen, Germany
Structure	Exoscope	Exoscope + microscope + endoscope	Exoscope	Exoscope	Exoscope
Focal length (mm)	200-500	170-260	-	220-550	200-450
Magnification	8–30, @ 300-mm WD	10×	12.5×	26×	10×
Additions	Navigation, hand grips	Navigation, hand grips, QEVO	Voice-activated control	Navigation, controller, foot switch	Hand grips, footswitch
Cost (in Dollars)	Approx 39,000	Approx 3,90,000	Approx 6,00,000-7,50,000	Approx 4,50,000	-
Robotic arm	Pneumatic arm 6-axis	Point-lock, position memory	Hands-free, position memory, 6-axis	Hands-free, 5-axis	Log-on-target, waypoints, position memory, 6-axis
Working distance (mm)	20-50 mm	200-625 mm	650 mm	220-550 mm	200-450 mm
Field of view (mm)	14–145, @ 200- to 500-mm WD, depending on zoom settings	-	6.5–207.9 mm	7.5 to 171 mm	70 - 130 mm
Stereopsis	3D, 4D	3D, 4D	2D, HD	3D, 4D	2D, 3D, HD
Illumination	LED	N/A	N/A	LED light, blue light adjustable	LED light, white/blue light adjustable, and simultaneous use
Features	ICG	ICG, flow assessments, fluorescence	Tractography	ICG, fluorescence, narrow-band imaging	ICG, fluorescence
Image capture	3D 4K monitor (3840 × 2160 pixels)	4K	-	4K monitor (4160 × 2160 pixels)	4K

VITOM 3D: Functioning as an extracorporeal visualization technology, the VITOM 3D system has demonstrated practical applications across various surgical disciplines. This system comprises critical components, including the operating telescope, camera, light source, holding arm, control unit, high-definition monitor, and documentation system, collectively forming the fundamental framework of VITOM 3D. It is an easy-to-use, 11 cm-long 0° telescope with integrated fiber optic light transmission that is also autoclavable. Its camera system features an integrated parfocal zoom lens on the 1 H-3Z full high-definition camera head. Cold light fountain made of 300 SCB XENON. It is linked to the telescope by a fiber optic connection [50].

The system incorporates a serial digital interface module with the IMAGE 1 HUB high-definition camera control unit SCB. This configuration features an extended, L-shaped articulated platform housing a singular mechanical center clamp, the securing mechanism for its five joints. The operation table is attached to the stand. An aiding surgeon can make height and varied angle modifications at different joints. The setup features a 27-inch full high-definition display with a maximum resolution of 1920 x 1080 and a 16:9 aspect ratio. High-definition digital imagery, videos, and audio recordings are systematically stored through a dedicated documentation system [50].

KINEVO 900 S: This system combines a microscope, an exoscope, and an endoscope (QEVO) into a single device, providing surgeons with the flexibility to choose their preferred visualization method at each stage of surgery. Each visualization mode has distinct benefits and drawbacks. With an expansive working distance of 625 mm, the system adeptly avoids interference with lengthy surgical tools used in spinal procedures. Furthermore, its capacity to be positioned significantly above the surgeon's line of sight while maintaining an unobstructed view of the monitors underscores its impressive flexibility. However, it is essential to consider that accommodating such a substantial working distance necessitates clearing the exoscope area. Further emphasizing the significance of keeping the space clear is the system's inclusion of unique features, such as robotic repositioning and point lock [51].

The Jackson spine table, also known as an operating table, is moved out from the center, typically where the light pendants were, to accomplish this. It is equipped with an attached robotic arm that supports point-lock and position memory. The system has a focal length ranging from 170 mm to 260 mm and a magnification factor of 10x. In addition to these specifications, the system offers supplementary functionalities, including indocyanine green imaging, fluorescence visualization, and flow assessment capabilities [51].

Modus V: This system is a fully robotic, hands-free, computerized 2D exoscope designed to enhance surgical precision and accuracy. Tracked surgical tools enable robotic camera movement and optical focal depth control without human control. The improved optics, featuring a 12.5 optical zoom, 10-μm resolution, and a working range of up to 65 cm, ensure a clear and natural view. Around the camera are four upgraded LED light sources. Cognitive optics integrated within the Modus V system can predict optimal lighting and camera conditions. This technology collaborates seamlessly with a 4K digital medical-grade monitor, ensuring a pristine view of the surgical field [52].

The Modus V incorporates a set of five robotic motions, each responsive to touch commands. These motions execute precise visual adjustments by recognizing and promptly enacting the desired manual modifications. Operating surgeons utilize their non-dominant hands to direct the guided suction tool to the specific region of interest. Subsequently, a robotic arm with a camera and light source autonomously tracks the guided suction, delivering clear and focused visualization of the targeted area [52].

ORBEYE: The system's foundation is a versatile and unrestricted arm that interfaces with the processor and cradles the dual-optics camera system in a secure position. Utilizing fiber optics, an LED light source is transmitted to the camera head. The visual output is projected onto a 31-inch 4K 3D monitor, boasting a resolution of 4160 x 2160 pixels. The impressive magnification potential of up to 26x is achieved through a 1:6 optical zoom, a 2x digital zoom, and the substantial magnification capacity of the expansive screen. Creating a 3D image is facilitated by using polarized 3D glasses in tandem with the technology [53].

The system also supports an auxiliary 2D output, enabling display on additional monitors within the operating theater to assist other team members. Furthermore, a secondary, smaller screen positioned directly behind the operating surgeon provides the assisting surgeon with a clear view, fostering enhanced collaboration and engagement throughout the surgical procedure [53].

Aeos: This 3D robotic digital microscope represents a cutting-edge 3D heads-up surgery system enhanced by robotic assistance. Key features of the system include a high-performance camera. This versatile 6-axis robotic arm enables flexible setup configurations, a 3D surgical screen with a wide 16:9 aspect ratio, and high dynamic range imaging capabilities, available in either full high-definition (1080p stereoscopic imaging) or 4K ultra high-definition resolution. Integral to the system are 3D glasses that enhance the immersive experience. Complementing these elements is a control screen boasting a 15.6" display size and touchscreen functionality. The microscope's foundational structure integrates pivotal features, including 3D recordings, video outputs, video inputs (capable of accommodating MRI or CT scans), USB connectivity, and DICOM compatibility. A wireless or cabled footswitch with programmable buttons and a joystick enhances the user interface [54].

Indications

**Figure 3:**
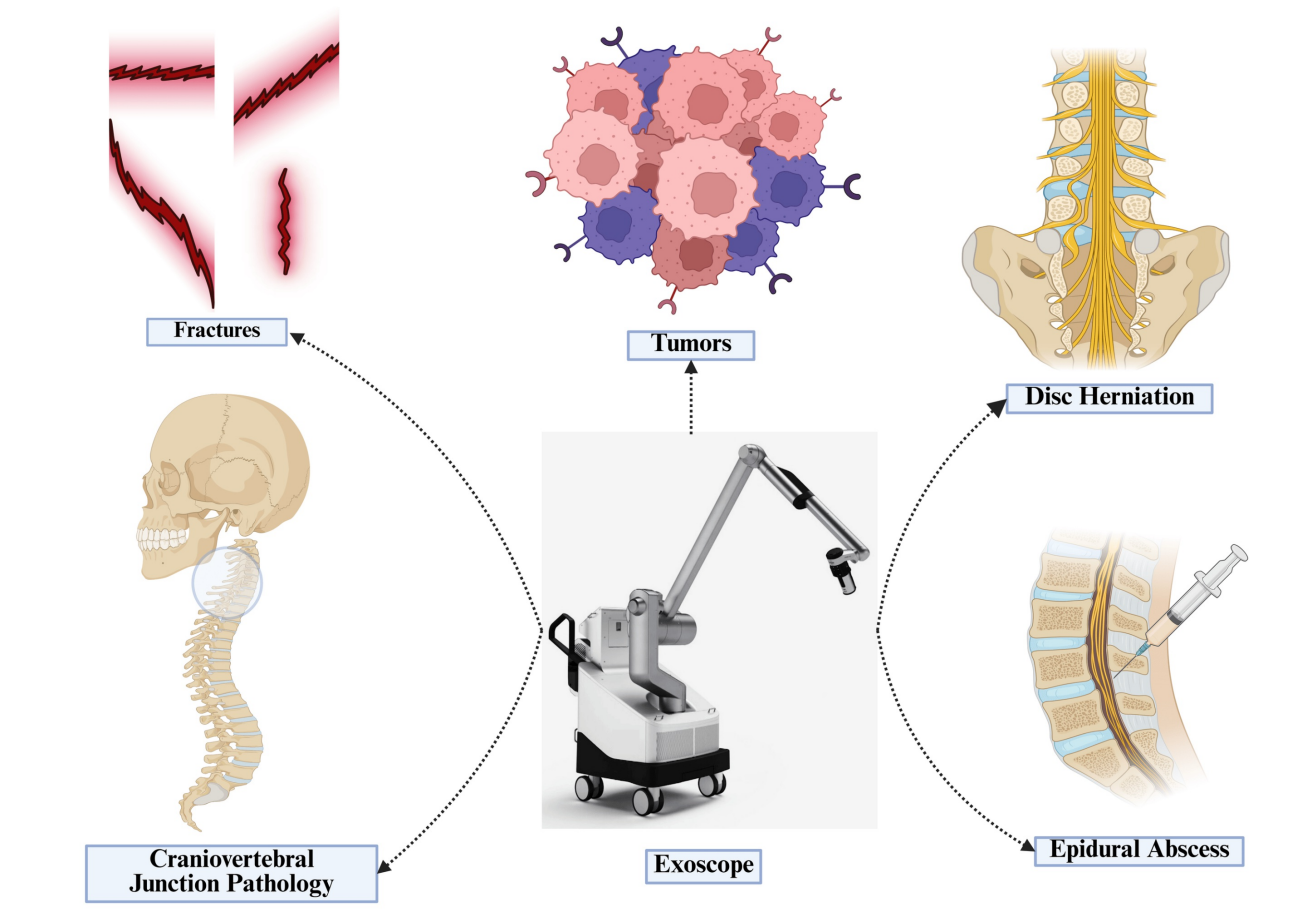
Use of exoscope in various conditions of the spine Image Credit: Authors. Created using BioRender.

Table 5 and Figure 3 comprise conditions and spinal procedures conducted through the exoscope application.

**Table 5:** Use of exoscope in various spine conditions/procedures

Spine procedures	Use of exoscope
	Anterior cervical discectomy and fusion [6,20,31-33,41,42,55-58]
	Transforaminal lumbar interbody fusion [41,57,39,30]
	Anterior lumbar interbody fusion [29]
	Oblique lateral interbody fusion [30]
	Cervical posterior decompression [30,31]
	Lumbar posterior decompression [6,32,40-42,45,58]
	Lumbar discectomy [33,41,55]
	Corpectomy [32,55]
	Cervical foraminotomy [42]
	Cervical laminectomy
Spine conditions	Neurofibroma [55], meningioma [44,55], angiolipoma [41], schwannoma [56]
	Metastasis [57,58]
	Disc herniation [31,44,56,57,58-60]
	Epidural abscess [33]
	Craniovertebral junction pathologies [61]
	Fractures

Ergonomics

The exoscope presents superior subjective ergonomics compared to the OM, which is often associated with less satisfactory ergonomics. With the exoscope, surgeons can maintain a comfortable and natural posture during surgery, whereas using the OM may require them to bend their necks and backs to look through an eyepiece. Furthermore, the exoscope helps reduce eye strain and fatigue experienced by the surgeon, which can be caused by prolonged use of the OM. However, it is worth noting that some studies have reported mild ocular discomfort, headaches, or nasal pain in a few participants who used 3D glasses with the exoscope [17,28,30,56,62,63]. However, we believe nasal pain can be mitigated by personalizing the 3D glasses according to individual surgeons [3,4].

As a result, the exoscope is associated with lower levels of surgeon discomfort compared to the moderate to high discomfort often reported with the use of the OM [30]. These ergonomic advantages of the exoscope contribute to improved surgical performance and overall satisfaction while reducing the risk of musculoskeletal injuries for the surgical team (Table 6) [3,4].

**Table 6:** Differences between OM and exoscope OM: operating microscope, 3D: three-dimensional, LED: light-emitting diode

	Exoscope	OM
Magnification range	1.9-39.3X	1-17X
Image quality	Excellent, surpasses OM	Standard, captured through an eyepiece
Depth of field	Longer than OM	Limited
Focal length	Longer than OM	Limited
Light source	LED	Xenon
Auto-focusing	Both have auto-focusing capability	Exoscope has a shorter refocusing time
Control mechanism	Footswitch and hand grip	Hand grip
Maneuverability	Highly portable and flexible	Occupies considerable space
Stereopsis	Offers 3D visualization	Offers depth perception
Education usefulness	Allows team and trainee viewing	Limited to eyepiece or small monitor
Recording and streaming	Yes, for teaching and research	May not have this feature
Ergonomics	Superior subjective ergonomics	Associated with less satisfactory ergonomics
Surgeon discomfort	Lower levels compared to OM	Medium to high discomfort with OM

Learning Curve

The adoption of any new technology or surgical procedure must consider the associated learning curve. It refers to the time and practice necessary for surgeons and the surgical team to become proficient and at ease when utilizing the technology. Given the relatively recent introduction of exoscopes in spine surgery, mastering this technique can be more challenging for less experienced surgeons. As the surgical team becomes acquainted with the equipment and improves their skills, the initial exoscope learning curve may lengthen the time of surgeries. The entire operating room team's learning environment and workflow are enhanced by decoupling the exoscope source and visual production, which promotes improved communication and instrument interchange. Additionally, there is significantly less of a "learning curve" than with traditional endoscopic and microscopic techniques, as the neurosurgeon can easily employ well-known, standard equipment [26].

The learning curve for exoscopes is lessened because most seasoned spine surgeons are already accustomed to utilizing optical microscopes. Although there is a lower learning curve for optical microscopes due to their familiarity, new surgeons and operating room staff still require training on using and positioning them. Compared to exoscopes, surgeons already skilled with optical microscopes may experience a shorter learning curve. Exoscopes' modern technology and 3D visualization may initially make learning more challenging, but with proper training and experience, this difficulty can be successfully overcome [26].

Outcomes

We demonstrate the potential advantages of the 3D exoscope in reducing bleeding through an element-by-element examination of intraoperative blood loss. With improved visualization, surgeons may be better able to locate and stop bleeding sources, resulting in improved hemostasis and less blood loss. The improved magnification and depth perception may reduce tissue stress, stop bleeding, and enhance clarity and precision. Fewer revision operations may result in patients losing less blood overall. Compared to open surgeries, minimally invasive techniques typically entail smaller incisions, resulting in less tissue damage and possibly less blood loss. In two-level transforaminal lumbar interbody fusion, 3D compared to OM, the exoscope significantly reduced surgical drainage output and postpartum hemorrhage content. Our results show that in a two-level TLIF, the 3D exoscope was related to a quicker drainage tube removal process, a smaller overall drainage fluid volume, and a reduced need for autologous blood reinfusion. Compared to the OM group, postoperative hemoglobin and hematocrit levels were higher, whereas total blood loss and visible blood loss levels were lower [17].

Comparison With OM

One of the main advantages of the exoscope is the higher magnification range compared to the OM. The exoscope has a magnification range of 1.9-39.3X, while the OM has a range of 1-17X [9]. This enables the exoscope to visualize delicate structures and details in the surgical field more effectively. Moreover, exoscopes exhibit excellent image quality, surpassing that of the OM [9,17]. The exoscope captures images on a 3D high-definition or 4K monitor, while the OM captures images through an eyepiece [9]. The monitor's adjustability to various angles and positions enhances the comfort and ergonomics of the surgeon and surgical team (Table 5).

The exoscope has a greater field depth and focal length than the OM [20,30,31,39,57]. This means the exoscope can capture a larger area of the surgical field in focus than the OM. The exoscope has a working distance of 300-1000 mm, which is longer than that of the OM [9], and can be advantageous for avoiding collisions with instruments and surgical staff. The exoscope utilizes an LED light source, whereas the OM employs a xenon light source. The LED light source is more energy-efficient and durable than the xenon light source. Both the exoscope and the OM have auto-focusing capabilities. This implies that both systems can autonomously adapt the image focus in response to instrument movement or changes in the surgical field. Notably, the exoscope outperforms the OM's quicker refocusing time [31,56]. This means that the exoscope can quickly restore the focus of the image after a change in depth or position, while the OM may take longer. This can reduce the need for manual adjustments and interruptions during surgery [3,4].

Both the exoscope and OM can be controlled using a foot switch and hand grip. The foot switch allows the surgeon to change the magnification, focus, zoom, and brightness of the image without using their hands. The hand grip allows the surgeon to move and rotate the exoscope or OM around the surgical field [34]. However, some surgeons may prefer one controller type over another, depending on their personal preferences and habits [3,4].

The exoscope offers enhanced maneuverability compared to the OM due to its compact size and lightweight design, making it highly portable and flexible. Unlike the OM, which occupies a significant amount of space, the exoscope takes up minimal space, providing improved accessibility and convenience in various settings and situations. Exoscopes offer a significant advantage in stereopsis, providing 3D visualization that enhances depth perception during surgery. However, it is worth noting that the exoscope and the traditional operative microscope provide depth perception [3,4].

The exoscope has higher educational usefulness than the OM [31]. It allows the surgical team and trainees to view the same image as the surgeon on a large monitor. In contrast, the OM only allows a limited number of observers to view the image through an eyepiece or a small monitor. The exoscope can also record and stream images for teaching and research purposes, whereas the OM may not have this feature. The exoscope can enhance communication and collaboration among the surgical team and trainees, while the OM may isolate the surgeon from the rest of the team [3,4].

Future directions

While exoscopes offer more promising visualization, versatility, safety, and ergonomic features compared to conventionally used surgical microscopes, surgeons often encounter maneuverability problems. Surgeons often reported a slow learning curve and the need for frequent readjustments to the camera apparatus [32,64]. Surgical exoscopes require precise rotational adjustments to acquire an appropriate orientation, which is often much more complex than OM [45,62]. These limitations highlight the need for a more hands-free control and visualization device, enabling surgeons to use both hands in the field. In keeping with the ever-evolving trends in medical technology, integrating exoscopes with robotics could be a promising development, enabling surgeons to leverage the potential of exoscopes fully.

Currently, the Modus V exoscope enables robot-assisted functions, including the ability for the robotic arm and light source to follow a suction tip, guided by the non-dominant hand, and autofocus on the tip, in addition to providing excellent 3D visualization of microanatomical structures [65,66]. While there are limited large-scale studies that analyze the potential and feasibility of this particular model, these features could help enhance surgeons' technological confidence and eliminate the burden of distractions during surgery. Furthermore, with the advent of AI, machine-learning models can be generated, which allow the manipulation of voice-assisted functions to create a novel, ultra-modern neurosurgical experience.

As technology evolves, we can expect exoscopes to become more compact, lightweight, and user-friendly, making them more accessible to a broader range of surgical settings. Integrating AI and machine-learning algorithms could enhance the capabilities of exoscopes, enabling features such as voice-assisted controls, automated image recognition, and even augmented reality overlays. These innovations have the potential to streamline procedures, shorten the learning curve for new users, and significantly improve surgical precision and outcomes.

Limitations

While our study aims to highlight the latest features of new-generation exoscopes, the rapid pace of technological advancements in neurosurgery may render the presented data less relevant and limited in scope. Additionally, the study was constrained by a lack of extensive literature analyzing recently developed exoscope models. The niche focus on spinal surgeries has allowed us to review most aspects of exoscopes comprehensively; however, due to the vast potential applications of exoscopes in other surgeries, some aspects may need to be addressed that could be better explored.

## Conclusions

Exoscopes offer a 3D high-definition digital camera system with enhanced ergonomics, increased mobility, improved collaboration with the surgical team, seamless integration with navigation systems, and enhanced accuracy. These advancements have led to better patient outcomes, reduced recovery times, and enhanced quality of life for individuals with spinal conditions and injuries. The ergonomic advantages of exoscope technology contribute to a healthier and more sustainable surgical practice. Additionally, using exoscopes in spine surgery offers higher educational usefulness than traditional OMs, allowing surgeons and trainees to view the surgical field on a large monitor and facilitating improved communication and collaboration. Adopting exoscopes in spine surgery is still in its early stages, and specific challenges must be addressed. Surgeons and surgical teams may initially face a learning curve, and the high procurement costs, along with limited availability of exoscope equipment in resource-constrained settings, may hinder widespread implementation. However, with technological advancements and increased familiarity with the equipment, these challenges are expected to lessen over time.
